# Heterogeneous Contributing Factors in MPM Disease Development and Progression: Biological Advances and Clinical Implications

**DOI:** 10.3390/ijms19010238

**Published:** 2018-01-13

**Authors:** Bhairavi Tolani, Luis A. Acevedo, Ngoc T. Hoang, Biao He

**Affiliations:** Thoracic Oncology Program, Department of Surgery, Helen Diller Family Comprehensive Cancer Center, University of California, San Francisco, CA 94115, USA; Luis.Acevedo@ucsf.edu (L.A.A.); Ngoc.Hoang@ucsf.edu (N.T.H.)

**Keywords:** malignant pleural mesothelioma (MPM), tumor microenvironment heterogeneity, molecular pathways, tumor suppressors, immunotherapy, developmental cell pathways

## Abstract

Malignant pleural mesothelioma (MPM) tumors are remarkably aggressive and most patients only survive for 5–12 months; irrespective of stage; after primary symptoms appear. Compounding matters is that MPM remains unresponsive to conventional standards of care; including radiation and chemotherapy. Currently; instead of relying on molecular signatures and histological typing; MPM treatment options are guided by clinical stage and patient characteristics because the mechanism of carcinogenesis has not been fully elucidated; although about 80% of cases can be linked to asbestos exposure. Several molecular pathways have been implicated in the MPM tumor microenvironment; such as angiogenesis; apoptosis; cell-cycle regulation and several growth factor-related pathways predicted to be amenable to therapeutic intervention. Furthermore, the availability of genomic data has improved our understanding of the pathobiology of MPM. The MPM genomic landscape is dominated by inactivating mutations in several tumor suppressor genes; such as *CDKN2A*; *BAP1* and *NF2*. Given the complex heterogeneity of the tumor microenvironment in MPM; a better understanding of the interplay between stromal; endothelial and immune cells at the molecular level is required; to chaperone the development of improved personalized therapeutics. Many recent advances at the molecular level have been reported and several exciting new treatment options are under investigation. Here; we review the challenges and the most up-to-date biological advances in MPM pertaining to the molecular pathways implicated; progress at the genomic level; immunological progression of this fatal disease; and its link with developmental cell pathways; with an emphasis on prognostic and therapeutic treatment strategies.

## 1. Introduction

Malignant mesothelioma is an aggressive and universally lethal cancer that arises because of pathological transformations in the mesothelium, a protective serous membrane that lines several organs in the body, such as the lungs (pleural), the intestines (peritoneum), the heart (pericardium) and tunica vaginalis. Of these, malignant pleural mesothelioma (MPM) is the most common and accounts for the predominant subtype—80% of all cases. MPM has an unusually dismal prognosis; its 5-year survival rate is a mere 10%, making it the most fatal among rare cancers [1]. Although MPM is classified as a rare disease and an estimated 3000 cases are diagnosed in the United States each year, the incidence is expected to remain steady, or rise, until 2055, because of the developmental latency of the disease; correspondingly, two thirds of mesothelioma cases are diagnosed in patients over the age of 65 [1]. The established cause of carcinogenesis is direct workplace exposure to asbestos, a naturally-occurring silicate fibrous mineral used in insulating material, and approximately 80% of MPM cases can be linked to it; consequently, males have a four times higher incidence rate than females [1]. While asbestos use has declined in the United States since the 1980s, it has not been banned, as in other countries; thus, more clinical diagnoses are expected to emerge as a result of the disease’s late manifestation (~20–40 years from exposure). Although the exact mechanism by which asbestos fibers lead to disease onset is still under debate, some hypotheses include the role of toxic oxygen radicals, elevated growth factor-induced cell signaling of kinases, and chronic inflammation, which ultimately leads to malignancy [2]. Other etiological factors include infection with simian virus 40 (SV40) and exposure to erionite and radiation, but their contributions remain controversial [3].

Current treatments for MPM are limited to surgery, radiation, and chemotherapy. However, since 80% of patients are diagnosed in stage III/IV, they are not candidates for surgical cure because their disease is no longer resectable [4]. Some hope lies in the recent advancement of intensity-modulated radiotherapy (IMRT), which precisely delivers radiation doses to the malignant tissues, while sparing normal counterparts [5]. Unfortunately, because of its low radio- and chemo-sensitivity, MPM remains unresponsive to systemic therapy. Currently, there are only two Food and Drug Administration (FDA)-approved chemotherapeutic drugs specifically for treating MPM: cisplatin and pemetrexed. Although combination therapy, consisting of cisplatin and pemetrexed, has shown promising prognostic outcomes and a response rate of 41.3%, making it the standard treatment for mesothelioma, overall survival has been a low 16.6 months [6].

Histologically, there are three main sub-types of malignant mesothelioma: epithelioid (~60% of cases), sarcomatous (~20%), and biphasic (combinations of the first two) [7]. Whereas patients diagnosed with epithelioid mesothelioma reportedly survive the longest (12–27 months), the appropriate course of treatment can be onerous to determine [8]. This is due to the inherent tumor heterogeneity and the difficulty in staging the disease, which underscores the need to better understand the disease pathobiology at the molecular level.

The goal of this review is to provide translational scientists with an up-to-date account of recent and potential therapeutics to address the treatment of MPM. We discuss current advances in mesothelioma biology and heterogeneity in the tumor microenvironment, starting with heterogeneity at (1) the molecular pathway level, followed by progress at (2) the genomic level, then (3) immunological progression of MPM, and finally the link with (4) developmental/stem cell pathways associated with the disease. In the first section on molecular pathways, we explicate translational implications of pathways, such as angiogenesis, apoptosis, and the cell cycle, along with their characteristics in mesothelioma. In the genomic landscape section, we explore the three main genetic alterations exclusive to this rare cancer. Next, we review the contribution of the immune environment, including the stromal compartment, immune population and tumor cells. The last section describes the development pathways, such as Hedgehog, Wnt/β-catenin, Notch and Hippo/yes-associated protein (YAP) in MPM. Our focus is to discuss promising new therapeutic strategies under investigative development, which could potentially permit a longer and better quality of life for those with mesothelioma.

## 2. Molecular Pathways in Malignant Pleural Mesothelioma

Therapeutic exploitation of the following vulnerabilities present in MPM, by harnessing overabundant growth signaling factors and cytokines, could help in the fight against this disease. A summary of the clinical study status and potential feasibility for the following are provided in Table 1.

### 2.1. Angiogenesis

The formation of new blood vessels via angiogenesis is prompted by the release of cellular cues, such as cytokines, and mesothelioma has been shown to express angiogenic factors along with their corresponding cellular receptors [9,10,11,12]. Approximately 30% of MPM cases express vascular endothelial growth factor (VEGF), and about 67% express the sub-type VEGF-C [11,12], one of the more abundant angiogenic factors found in any solid tumor patients [10]. Moreover, about 20% of cases show positive staining for the VEGF receptor (VEGFR-1), expressed almost exclusively in endothelial cells [11]. Inhibition of VEGF or its receptors can decrease proliferation in mesothelioma [10]. Other angiogenesis-related factors, such as fibroblast growth factor-2 (FGF-2) and hepatocyte growth factor (HGF), are hyper-expressed in mesothelioma, at 50% and 85%, respectively [12,13]. HGF, known to induce angiogenesis via activating endothelial cell migration and capillary tube formation, is significantly associated with epithelioid histology [13]. Given the strong correlation of elevated angiogenesis levels with diminished survival of MPM patients, several anti-angiogenic agents have been under clinical investigation. Bevacizumab, a neutralizing monoclonal antibody, targeted at VEGF-A, received FDA approval in 2004 for use in colon cancer [14]. Although it led to considerable toxicity in clinical trials, investigations have shown that the addition of bevacizumab to cisplatin and pemetrexed combinations was beneficial in MPM [15].

The strong rationale for angiogenesis inhibition has prompted the study of several alternative small molecules in MPM, such as vatalanib, lenvatinib, thalidomide, sorafenib and sunitinib, as well as other modalities, such as a competent adenovirus and NK-4, which is an HGF variant. A clinical trial was launched to investigate the efficacy of vatalanib, a dual pan-VEGFR and platelet derived growth factor receptor (PDGFR)-β inhibitor in mesothelioma, and although the results showed minimal benefits, its low toxicity could warrant further experimentation to explore synergistic effects if combined with other standard treatments [16]. Lenvatinib is a pan-tyrosine kinase inhibitor, aimed at multiple targets, such as VEGFR-2, fibroblast growth factor receptor (FGFR), and PDGFR, which inhibits endothelial cell growth, crucial for supplying blood to tumor cells [17]. Thalidomide, sorafenib and sunitinib are in various phases of clinical investigation [18,19,20] and a replicative competent adenovirus that targets the VEGF promoter is being tested preclinically [21]. NK-4, produced during inflammation, is a mimic fragment of HGF that can bind to its receptor, c-Met, without angiogenic stimulation, and therefore, when injected as an adenoviral vector into mesothelioma-bearing mice, there was tumor growth inhibition, caused by decreased blood vessel formation and apoptosis induction [22]. Finally, additional anti-angiogenesis agents under clinical investigation that target VEGFR, some of which are being combined with chemotherapy, include dovitinib, nintedanib, and cediranib and others [23].

### 2.2. Apoptosis

Programmed cell death, or apoptosis, is a carefully regulated process evaded by most cancers, partly because of inactivating mutations in the tumor suppressor gene, *TP53*, which is found to be mutated in about 50% of human tumors [24]. Distinct from other cancers, mesothelioma typically expresses functional p53 [25] and only 20–25% of cases harbor *TP53* mutations [26]; thus, both p53-dependent and independent mechanisms of apoptosis occur in MPM. Mesothelioma cell lines with normal p53 are sensitive to cisplatin treatment, show increased DNA binding and elevated phosphorylation, which prevents targeted degradation by MDM2, and thus, increased transcriptional activation of apoptotic genes ultimately leads to p53-dependent cell death [27,28]. Survivin, a negative regulator of p53, is an anti-apoptotic protein associated with unfavorable patient outcomes [28] and as a treatment strategy, introduction of inhibitory anti-sense *Survivin* oligonucleotides sensitized mesothelioma cells to chemotherapy and induced apoptosis [27,28].

Downstream of p53, many defects in the apoptotic core machinery have been reported in cancer. The Bcl-2 family of proteins is most well-known for orchestrating mitochondrial-mediated apoptosis and encodes both pro- and anti-apoptotic proteins that either protect tumor cells from chemotherapy-induced apoptosis or confer resistance to these drugs. One apoptotic repressor, Bcl-xL, which is strongly expressed in mesothelioma, prevents apoptosis induction by inhibiting mitochondrial permeabilization and activated caspase release, thus contributing to tumor growth [26]. Small molecule HDAC inhibitors and antisense oligonucleotides directed at Bcl-xL were shown to induce tumor cell death via apoptosis and enhance chemo-sensitivity in MPM [29,30]. A second anti-apoptotic protein, MCL1, is also expressed in mesothelioma, and together with Bcl-xL, can further inhibit apoptosis and confer resistance to mesothelioma [25]. Bcl-2 is not commonly expressed in mesothelioma, but down-regulating Bcl-2 in conjunction with Bcl-xL is more effective in inducing apoptosis [25]. One such small molecule, 2-methoxy antimycin A3, alone, or in chemotherapeutic combinations, exhibited a synergistic relationship in promoting tumor cell death via apoptosis, as well as promoting chemo-sensitivity [31].

Outlined below are several noteworthy proof-of-concept studies, which demonstrate that the induction of apoptosis in resistant mesothelioma cells could be used as a therapeutic approach. These comprise taking aim at the following targets: focal adhesion kinase (FAK) and Src, Fas receptor and modulation of reactive oxygen species (ROS), calcium ions, and tumor necrosis factor-related apoptosis inducing ligand (TRAIL).

#### 2.2.1. FAK (Focal Adhesion Kinase) and Src, Fas Receptor and ROS (Reactive Oxygen Species)

FAK is an important regulator of cell proliferation, migration, and invasion expressed in primary mesothelioma cell lines. Dual pharmacological inhibition of FAK and MDM2 was synergistic and resulted in decreased cell viability and increased total phosphorylation of p53 and p21, along with a decrease in the proliferative marker, cyclin A [32]. Furthermore, about half of the mesothelioma samples express a protein similar to FAK called Src, whose presence is correlated with advanced tumor pathology given its role in cell migration and invasion. The use of the Src/Abl inhibitor, dasatinib, decreased activated Src in mesothelioma cell migration and invasion, which led to cell cycle arrest and apoptosis in sensitive cell lines [33]. The Fas family of receptors and ligands are part of many death-signaling pathways that can initiate apoptosis. One study showed that the combined effect of cisplatin-induced ROS levels with Fas ligands (FasL) and sensitized Fas receptor positive cells to apoptotic death by activating caspase 9, evidenced by apoptosis-specific DNA fragmentation [34]. These in vitro data were recapitulated in an in vivo mouse model, where tumor growth decreased significantly after treatment with cisplatin and FasL, thereby highlighting the possibility for personalized treatment of Fas-positive patients by combining ROS-mediated apoptosis induction with chemotherapy [34].

#### 2.2.2. Calcium Ions

Mitochondrial calcium ion concentration can induce death via apoptosis in cancer cells and tissue. When exogenous calcium ions were added to primary mesothelioma culture, there was an increase in apoptosis via cleaved caspase 3 activities, which also occurred if the mitochondrial calcium ion uniporter was expressed exogenously, underscoring the utility of harnessing this mechanism for MPM treatment [35].

#### 2.2.3. TRAIL

TRAIL can induce apoptosis by binding to death receptors expressed on cancer cells. Immune modulating human mesenchymal stromal cells (MSCs) have been genetically engineered to express TRAIL, which, when injected intraperitonially into mesothelioma-bearing mice, significantly reduced tumor burden [36]. This was accompanied by an increase in apoptotic, Terminal deoxynucleotidyl transferase dUTP nick end labeling (TUNEL)-positive cells, which potentially allows for personalized and autologous treatment of mesothelioma by harvest and modification of patients’ own MSCs to express TRAIL without adverse immune reactions [36]. 

### 2.3. Cell Cycle Effectors

Uncontrolled cell growth results in cancer when mutations accumulate in several control mechanisms that cause cells to no longer respond to biological signals. The effectors of the cell cycle machinery play crucial roles in the progression of MPM and a study that compared patients by grouping long-term (>1 year) and short-term (<1 year) survivors reported that the majority of cell cycle genes, such as *AURKA*, *AURKB*, *CDC25C*, *PTTG*3, *CCND1*, and *KIF4*, are negatively correlated with survival [37]. Christensen et al. demonstrated that several cell cycle genes—*APC*, *CCND2*, *CDKN2A*, *CDKN2B*, *APPBP1*, and *RASSF1*—are methylated in older mesothelioma patients, and those with methylation of *RASSF1*, a gene that encodes an activator of G1/S cell cycle arrest, through sequestering of CCND1, is significantly associated with elevated asbestos body counts and therefore longer asbestos exposure [38]. These investigators also showed a significant correlation between asbestos body count/asbestos exposure and the number of cell cycle genes that are methylated, highlighting the importance of age and the duration of asbestos exposure in the development of mesothelioma [38].

Counterintuitively, an important regulator of DNA damage-mediated cell cycle arrest, checkpoint kinase 1 (CHEK1), is overexpressed in 50% of mesothelioma cases compared to normal pleural tissue, and *CHEK1* knockdown leads to synergistic apoptosis in mesothelioma cell lines when combined with the chemotherapeutic drug, doxorubicin [39]. In addition, over 70% of mesothelioma cases express YAP, which is known to up-regulate cell cycle genes in mesothelioma and subsequently decrease the Merlin–Hippo interaction. Given that YAP promotes TEA domain family member 1 (*TEAD*)-mediated transcription of cell cycle genes, such as *CCND1* and *FOXM1*, one study found that silencing *YAP* in mesothelioma resulted in suppression of these genes and inhibition of cell motility, invasion and anchorage-independent growth [40]. Concurrently, inhibition of YAP led to decreases in *E2F1*, *AURKB*, *PLK1*, *NEK2*, and anti-apoptotic *BIRC5*/*Survivin*, as well as an increase in pro-apoptotic *BCL2L11/BIM*, which ultimately resulted in broad decreases in anchorage properties, proliferation, migration, and invasion [40]. Thus, targeting YAP could potentially be feasible in treating this aggressive cancer.

### 2.4. TERT

In addition to cell cycle regulators and effectors, telomere production and regulation are also dysfunctional in mesothelioma [41]. For a tumor cell to continue to grow and proliferate, its telomere length must be elongated to ensure the basal integrity of its already unstable genome after many rounds of cell division. Tallet et al. established that telomerase activity is present in over 90% of mesothelioma cases, and its catalytic subunit *TERT* mRNA is expressed in over 80% of patient samples. While *TERT* can be mutated at its promoter region (>15% of cases present with C228T somatic mutations), the *TERT* locus (5p15.3) can also be amplified to induce higher *TERT* levels (around 50% of mesothelioma); however, the *TERT* promoter mutation is responsible for elevated *TERT* expression. Mutations in the *TERT* promoter along with *NF2* and *CDKN2A* alterations frequently occur concurrently and anti-telomerase drugs might therefore have clinical utility when combined with other targeted therapies [42,43].

### 2.5. Growth Factors Implicated in Mesothelioma

Several growth factors involved in MPM have been identified, such as epidermal growth factor receptor (EGFR), platelet derived growth factor receptor (PDGFR), and fibroblast growth factor (FGF), making them potential candidates for therapeutic intervention.

#### 2.5.1. EGFR

Although mutations that lead to overexpression of EGFR have been associated with a wide range of malignancies, the role of elevated levels of EGFR in MPM appears limited. Okuda et al. showed that 0 out of 25 MPM samples expressed any of 13 known EGFR mutations, and only eight were positive for the presence of EGFR via immunohistochemistry (IHC) [44]. Although another study reported higher rates of EGFR expression in MPM tumors using IHC (38/83 positive for EGFR) [45], both studies confirmed by fluorescence in situ hybridization (FISH) analysis that there are only few occurrences of EGFR copy number gains, and thus EGFR overexpression did not appear to be due to gene amplification [44,45]. Whereas growth in EGFR-expressing mesothelioma cell lines is significantly deterred by EGFR small molecule inhibitors [45], clinical evaluations of gefitinib and erlotinib were dismal in MPM patients [46,47]. Only two mesothelioma patients out of 43 responded to gefitinib and 21 had stable disease, suggesting that a subset of patients might benefit from this treatment [46].

#### 2.5.2. PDGFR

PDGFR has two subtypes: alpha (α) and beta (β); PDGFRα can bind three different PDGF ligand combinations (AA, AB, and BB), but PDGFRβ only binds to PDGF-BB [48,49]. Whereas PDGFRα functions in normal mesothelial cell growth, PDGF-BB and PDGFRβ are implicated in MPM growth [48,49]. Like EGFR, PDGFRβ is overexpressed in 20–40% of MPM samples without frequent somatic mutations, and overexpression of PDGFRβ levels correlates with poor survival [44]. Additionally, human MPM cell lines have shown up to 70-fold increases in PDGFRB expression, when compared to a non-malignant mesothelial cell line [49]. The use of si*PDGFRB* to silence gene expression in these MPM cell lines caused a significant reduction in growth and clonogenicity [49]. Since non-malignant Met5A cells showed no noticeable adverse effects after *PDGFRβ* silencing, it could be a potential target gene for therapeutic intervention [49].

#### 2.5.3. FGF

Hyper-expressed components of the FGF pathway contribute to MPM tumorigenicity and in particular, FGF2, FGF18, and FGFR1 have shown elevated levels in MPM cell lines and human tissue samples [50,51]. Schelch et al. demonstrated that FGF2-mediated stimulation increased MPM cell proliferation and motility but did not affect normal mesothelial controls [50], establishing the importance of FGF’s role in tumorigenicity and thus the potential for therapeutic interference as a treatment route. Furthermore, high serum and pleural effusions of FGF levels are significantly associated with poor survival [52]. Use of the FGFR1 inhibitor, PD-166866, was particularly promising as it not only hindered in vitro MPM cell proliferation and caused an increase in death via apoptosis, but also reduced in vivo MPM tumor growth in mouse models; this was accompanied by synergism when coupled with cisplatin or radiotherapy treatment [50]. Moreover, Pattarozzi et al. reported that pharmacological inhibition with sorafenib reduced tumor growth in MPM primary cells, mainly by derailing FGFR1 activation [53]. Despite previous limited success with sorafenib in clinical trials, this study renews interest in sorafenib treatment in select MPM patients who have particularly highly active FGFR1. Similarly, the tyrosine kinase inhibitor (TKI) ponatinib has also been shown to selectively inhibit growth and clonogenicity of MPM cell lines that express FGFR1 [51], further suggesting its potential for targeted therapy in MPM.

## 3. Genomic Landscape Prevalent with Tumor Suppressor Inactivation

**Figure 1:**
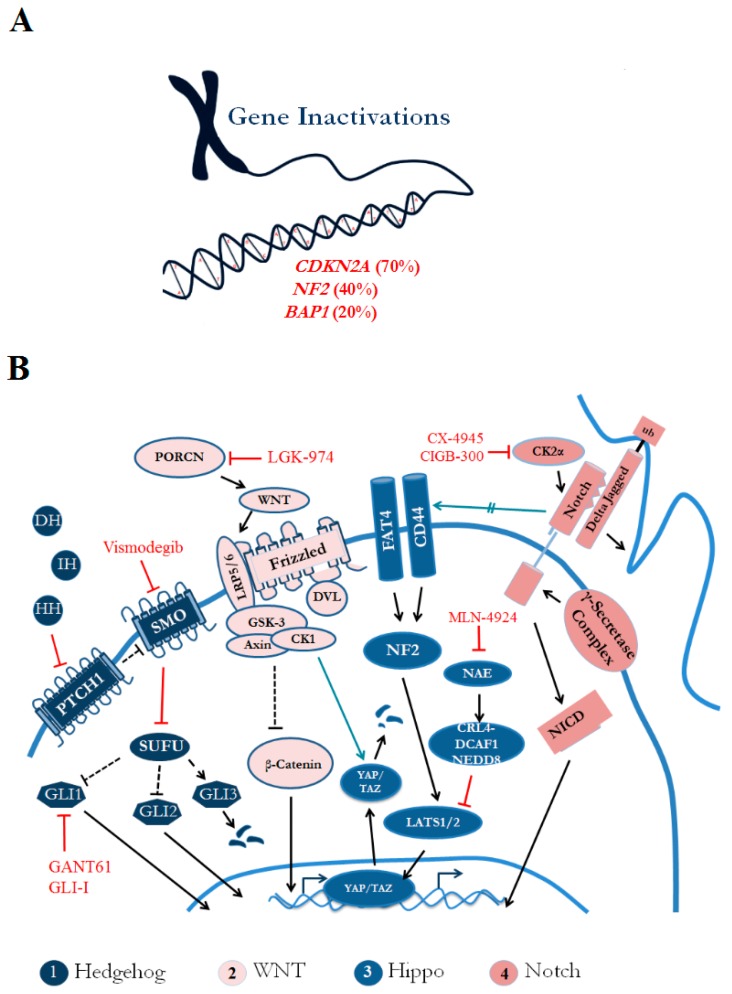
Heterogeneous contributing factors in MPM disease progression. (**A**) The MPM genomic landscape is dominated by frequent gene inactivation in *CDKN2A*, *NF2* and *BAP1*. (**B**) Several stem cellular signaling pathways, such as Hedgehog (Hh), Wnt/β-catenin, Hippo/YAP and Notch have been implicated in MPM pathobiology. (1) Hh ligands (DH, IH, and HH) bind patched (PTCH1) to relive inhibition of Smoothened (SMO) and this activates the Hh signaling pathway, via transcriptional activation of glioma-associated protein (Gli) factors; in the inactive state, Gli3 gets degraded when suppressor of fused homolog (Sufu) is inhibited. (2) Protein-serine O-palmitoleoyltransferase porcupine (PORCN) is required for efficient binding of Wnt ligands to cell-surface Frizzled (Fzd) receptors and to LRP5/6 which signals to the Dishevelled (Dvl) proteins. This causes an accumulation of β-catenin in the cytoplasm, followed by nuclear translocation and activation of transcription factors. (3) Hippo signaling consists of a cascade of kinases, which ultimately phosphorylate and activate serine/threonine-protein kinase (LATS)1/2 to inhibit two major downstream effectors of the Hippo pathway—YAP and Tafazzin (TAZ)—to stymie signaling (protein degradation). However, in *NF2*-mutant tumors, NAE activates CRL4^DCAF1^ and thus sustains tumor growth. (4) The Notch pathway can be activated by CK2α and when the Notch transmembrane receptors bind to a Notch ligand, a cascade of events promote gene transcription via γ secretase. Therapeutics (red) to target these pathway components have been developed for Smoothened (Vismodegib), Gli (GLI-I, GANT61), PORCN (LGK-974), NAE (MLN-4924) and CK2α (CX-4945 and CIGB-300). Arrows indicate interaction or activating effect and T-bars indicate inhibition. Green arrows indicate cross-talk between pathways.

The genomic landscape of mesothelioma is characterized by frequent alterations in tumor suppressor genes resulting from mutational events involving copy number losses, single nucleotide variants (SNVs), gene fusions and splicing events. MPM exhibits a distinct genomic signature, including inactivating mutations in *cyclin-dependent kinase inhibitor 2A* (*CDKN2A*), and more recently, *ubiquitin carboxyl-terminal hydrolase* (*BAP1*) and *neurofibromin 2* (*NF2*), have been shown to be the most prevalent in this type of cancer (Figure 1A). Of particular interest, although the frequency of point mutations in cancer genes is low, several studies report global tumor suppressor inactivation, associated with CpG promoter methylation in MPM, which contrasts with the epigenetic landscape of normal pleura [54,55].

### 3.1. CDKN2A

Occurring at a frequency of over 70%, the most common homozygous deletion or incidence of gene inactivation in MPM is found in the 9p21 region of *CDKN2A*. As a result of alternative reading frames, this tumor suppressor gene acts as a negative regulator of cell proliferation, by encoding two distinct protein products: INK4A (p16, named by its molecular weight) and ADP-ribosylation factor (ARF) (human: p14, murine: p19). P16 is an inhibitory protein that prevents cyclin-dependent kinases, such as CDK4 and CDK6, from hyper-phosphorylating tumor suppressor protein RB, thereby activating RB, which blocks cell cycle progression from traversing the G1-S phase transition [26]. Inactivating mutations can thus disrupt this cell growth control pathway and contribute to carcinogenesis. Incidentally, P16 is also a key regulator of RB-mediated cellular senescence [56]. As part of the p53 pathway, P14 promotes ubiquitylation and degradation of MDM2, but loss of P14 increases MDM2 stability and activation, thereby repressing p53 and disrupting cell cycle control [26].

Using genetically engineered mouse models, Altomare et al. established the importance of losses in both *P16^INK4a^* and *P14^ARF^* in the latency and development of mesothelioma tumorigenesis. When exposed to asbestos, mice with mono-allelic deletions of *P16^INK4a^* or *P19^ARF^* (human: *P14^ARF^*) developed mesothelioma earlier and more often than their wild type littermates, and those with deletions of both *P16^INK4a^* and *P19^ARF^* showed even faster progression into malignancy relative to the mice bearing single deletions [57]. Furthermore, adenoviral-based reintroduction of *P16^INK4a^* or *P14^ARF^* showed reversal of mesothelioma growth by decreasing hyper-phosphorylation of RB, increasing p53 levels, and ultimately resulted in cell cycle arrest [43]. Interestingly, over 75% of patients with mesothelioma have only *P16^INK4a^* deletions, and these patients generally have poor prognoses and shorter survival rates; almost 30% of primary tumors have been reported to have methylated *P16^INK4a^* [26,58]. Moreover, sustained *P16^INK4a^* expression is associated with better survival in patients after chemotherapy, regardless of histological subtypes, and deletion of *P16^INK4a^* correlates with poor outcomes [37,59].

Pharmacological intervention, through the use of CDK inhibitors, is an attractive therapeutic strategy and a multicenter phase II clinical trial as a second-line treatment for MPM is ongoing [60].

### 3.2. NF2

Deleted in around 35–40% of MPM cases and often functionally inactive if present due to somatic mutations, the tumor suppressor, Neurofibromin 2 (*NF2*) gene, has been implicated as a “gatekeeper” in asbestos-induced mesothelioma tumorigenicity via several mechanisms [61,62,63,64]. First, it is a putative regulator of the Hippo/SWH (Sav/Wts/Hpo) signaling pathway, known to be important for tumor suppression, and encodes the ezrin radixin and meosin (ERM) family protein, Merlin. *NF2*-mediated oncogenesis stems from the loss of Merlin and subsequent dysregulation of the Hippo pathway [62,63]. Whereas the Hippo pathway typically halts cell growth and inhibits the transcriptional regulator, YAP, the lack of Merlin leads to overactive YAP and thus uncontrolled cell growth and cancer progression. By transducing *NF2* into MM cells that had *NF2* mutations, YAP has been shown to be translocated from the nucleus to the cytoplasm [63], suggestive of NF2’s role in YAP activity. In addition, YAP1 is negatively regulated by large tumor suppressor kinases (LATS1 and LATS2), which are commonly deleted in mesothelioma; in contrast, forced expression of LATS2 inactivates YAP1 and causes a reduction in cell growth [65].

Second, Merlin has also been suggested to negatively regulate the PI3K-AKT-mTOR signaling pathway, whose activation contributes to the pathogenesis of a number of malignancies [62,63]. In tumors that lack Merlin, the use of mTOR inhibitors led to reduced proliferation, yet were ineffective in Merlin-expressing tumors [62], suggestive of the role of the *NF2* deletion in MPM oncogenesis and its association with mTOR. *NF2*-depleted mesotheliomas are sensitive to the mTOR inhibitor molecule, rapamycin, and thus, a better understanding of the role of *NF2* in MPM, may hold pronounced potential for various targeted therapies.

Third, Cho et al. reported that NF2 typically prevents p53 inhibition caused by Snail-p53 interaction, but since it is often deleted in MPM, the tumor suppressive role of p53 is hindered, despite its low mutational frequency in MPM [25,26,61,66,67]. In MPM cell lines, a chemical inhibitor of Snail-p53 binding, GN25, induced p53 activity, followed by apoptosis, opening up potential therapeutic exploitation now that a clearer understanding of the molecular function of NF2 has been discerned [61]. GN25 is currently a drug candidate for MPM treatment, but there are no records of clinical trials at this time.

### 3.3. BAP1

The deubiquitinase enzyme encoded by the *BAP1* tumor suppressor gene is inactivated in over 60% of mesothelioma cases and is associated with global methylation via activation of PRC2. Rare germline mutations in *BAP1* contribute to predispositions in malignant mesothelioma, among other cancers, despite lack of exposure to high levels of asbestos or other carcinogens [68], suggesting that *BAP1* may serve as a predictive biomarker. Bott et al. reported that *BAP1* also harbored nonsynonymous mutations in 12/53 samples, was located in the center of the 3p21.1 locus, and also had somatic mutations and copy number losses [66]. Although they found no significant correlation between *BAP1* mutations and MPM subtype, Yoshikawa et al. found that *BAP1*-inactivating mutations occurred in epithelioid-type MM nearly exclusively, indicating that BAP1 may be more useful in diagnosing only epithelioid-type MM [67]. BAP1 plays a key role in chromatin biology by mediating deubiquitination of core histones and is recruited to double-strand breaks in DNA, given its role in DNA repair. Accordingly, *BAP1* inactivation results in impaired DNA repair functions, which can have dire consequences, consistent with its role as an epigenetic modulator and transcriptional regulator [69]. Thus, drugs designed to target the epigenome, and, in particular, enhancer of zeste homolog 2 (EZH2), may hold promise for MPM harboring *BAP1* mutations, but these have not been evaluated clinically.

## 4. Immune Microenvironment of MPM Tumors

The immune system plays a crucial role in tumor surveillance and attack; consequently, understanding the dynamic associations between pro-tumorigenic and anti-tumorigenic components of the MPM immune microenvironment is paramount to developing new treatments. Asbestos exposure by inhalation has become incontrovertible in MPM pathobiology and the physiological response of chronic inflammation as an immune reaction has been implicated in disease progression [70]. The secretion of VEGF and inflammatory cytokines by activated macrophages, coupled with the inability of mesothelial cells to expunge asbestos, along with the elevated presence of growth factors, results in malignant transformation. This leads to an influx of immune-suppressing cells like tumor-associated macrophages (TAMs), myeloid-derived suppressor cells (MDSCs) and regulatory T cells (T_regs_) [71]. These events further propel tumor growth, due to immune surveillance escape, influx of stromal fibroblasts, and an increase in angiogenesis-sustaining endothelial cells [71]. Deciphering what differentiates one tumor from another could help in modulating the immune microenvironment to effectively induce an anti-tumor immune response, by strategic design and delivery of appropriate therapeutic agents.

Broadly, the microenvironment of the mesothelioma tumor contains a heterogeneous network of stromal cells, the immune population, tumor cells, fibroblasts, extracellular matrix and blood vessels [72]. Here, we focus on aspects of the tumor microenvironment where more information is available.

### 4.1. The Stromal Compartment

Connective tissue-derived stromal cells in the body secrete growth factors, like hepatocyte growth factor (HGF), that support regular cell division, but their interaction with cancer cells can be deleterious as they provide an extracellular matrix for tumor progression. Cancer-associated fibroblasts (CAFs) and myeloid cells are both stromal cells that help malignant cells to proliferate, migrate, and offer resistance to therapy, primarily by preventing immune T cells from entering the tumor [73]. In turn, the tumor cells themselves produce cytokines, such as fibroblast growth factor 2 (FGF-2) and platelet derived growth factor (PDGF), to continuously recruit more fibroblasts, and then these fibroblasts generate even greater amounts of HGF, thus contributing to the growth of the tumor mass [73]. This symbiosis between tumor cells and their stromal neighbors has been demonstrated by co-culturing lung fibroblasts with mesothelioma cell lines or patient-derived fibroblasts, because proliferation and migration of cancerous cells increased significantly due to activated MET-induced HGF; these effects were abrogated when the cells were treated with an anti-HGF antibody or FGFR/PDGFR inhibitors [70]. Thus, targeting fibroblast activation protein (FAP) in stromal cells has led to a decrease in mesothelioma tumor growth in preclinical animal studies [74]. Since chimeric antigen receptor (CAR)-T cell therapy is still quite new and exciting, it remains to be seen whether FAP targeting will become an effective therapeutic.

On the other hand, myeloid cells, which are highly abundant in mesothelioma, can be both immunosuppressive, as well as immunostimulatory, which can lead to tumor cell evasion or immune activation, depending on the environmental cues present. The tumor microenvironment has been demonstrated to be generally more immunosuppressive because of programmed death-ligand 1(PDL1) upregulation, which can inhibit T cell function [75]. Methods to target PD1/PDL1 using nivolumab are currently in phase II clinical trials for MPM use, and this may be adopted for clinical practice in the future. However, given variations in the tumor microenvironment from one mesothelioma patient to the next, an analysis of all individual cells and factors which make up this complex ecosystem is important to the pursuit of treatment.

### 4.2. The Immune Population

The presence of several different types of immune cells which support tumor formation can have a clinical impact on the immune microenvironment of MPM patients. CD3^+^ pan T cells, CD4^+^ helper T cells, and CD8^+^ cytotoxic T cells are mainly found within the stromal compartment, CD25^+^CD4^+^FOXP3^+^ regulatory T cells border the tumor cells, and CD68^+^ macrophages and CD16^+^ natural killer cells make up the majority of the tumor infiltrating population within the tumor [72]. The presence of CD8^+^ cytotoxic T cells within the tumor has been associated with early stage disease, more apoptosis, and better survival. Conversely, in preclinical studies, the presence of immunosuppressive CD4^+^CD25^+^FOXP3^+^ regulatory T cells and immunosuppressive soluble factors inside the tumor corresponded with poor prognosis in MPM, due to suppression of anti-tumor immune responses [76]. Accordingly, Hegmans et al. demonstrated that survival increased when FoxP3^+^CD4^+^CD25^+^ T_regs_ were depleted in an in vivo model of MPM [72]. Adenosine and prostaglandin E2 (PGE2) immunosuppression has been shown to inhibit T cell function [77], and cyclooxygenase-2 (COX-2) suppression blocks the growth of mesothelioma tumors via an immunological mechanism which permits more effective cytotoxic T cell (CTL) build up in the tumors [78]. Kiyotani et al. demonstrated that different regions of the tumor yield different repertoires of tumor-infiltrating lymphocytes (TILs). This is due to the presence of distinct somatic mutations in different areas of the tumors, leading to discrete clonotypes of T cell receptors, and ultimate results in pronounced region-specific TILs [79]. The accumulation of CD8^+^ tumor infiltrating lymphocytes (TILs) in tumors of MPM patients who underwent surgical resection correlated significantly with better survival [80]. Dendritic cells are rare, and interestingly, Cornwall et al. showed that there was a decline in the dendritic cell population, a reduction in expression of CD68, which can affect the cell’s ability to become fully active, as well as a decrease in the cell’s antigen-processing ability, in mesothelioma patients, compared to age-matched healthy donors [72,81]. Eosinophils, neutrophils, mast cells, and B cells are also rarely observed [72]. Thus, the presence of unique cell surface markers found on these various T cell populations could be exploited to selectively target those populations which are implicated in MPM progression, but progress has been limited thus far.

### 4.3. Tumor Cells

Cancer cells found within the tumor secrete high levels of VEGF to recruit endothelial cells for angiogenesis. The formation of new blood vessels has been established to contribute to tumor development by providing a continuous source of nutrients, growth factors and other related molecules described here. Tumor cells are reported to have elevated levels of chemokine (C-X-C motif) ligand 1 (CXCL1), a chemokine that plays a role in inflammation and tumorigenesis, as well as interleukin-6 (IL-6), a cytokine that prevents immune dendritic cell development [72]. Similar to their symbiotic relationship with stromal fibroblasts, tumor cells also have a beneficial relationship with the tumor-infiltrating macrophages. Cornelissen et al. demonstrated that factors secreted by the tumor cells transformed normal macrophages into malignant M2 macrophages, which reciprocally produce cytokines, such as interleukin-1 (IL-1), interleukin-6 (IL-6), interleukin-10 (IL-10), VEGF, and transforming growth factor beta (TGF-β), which contribute to tumor formation and development [82]. Chronic inflammation of the stromal compartment is associated with better patient survival in patients with epithelial histology, regardless of whether they received prior neoadjuvant therapy [83]. Cutting off the supply of one or more of the above-mentioned factors in the surrounding milieu is a therapeutic strategy being used to combat MPM tumors.

## 5. Developmental Pathways in Malignant Pleural Mesothelioma

MPM tumorigenesis is linked with asbestos exposure, chronic tissue inflammation, and subsequent tissue repair. Tissue regeneration and repair is associated with stem cell renewal genes and stem cell pathway activation. The activation of stem cell signaling is generally regulated and chronic stimulation and accompanying oncogenic events in these developmental pathways are correlated with poor prognosis in MPM patients [84]. Below we describe the role of four stem cell-associated developmental pathways (Figure 1B) and how they contribute to the heterogeneity in MPM tumors.

### 5.1. Hedgehog Pathway

Upon Hedgehog (Hh) ligand binding, the inhibition of transmembrane protein, Patched on Smoothened (Smo), is relieved and this activates the Hh signaling pathway via transcriptional activation of glioma-associated protein (Gli) factors [85]. Hedgehog-dependent Gli-mediated transactivation of target genes is negatively regulated by suppressor of fused homolog (Sufu); it is required for normal embryonic development and its loss leads to unfettered Hh activation and lethality [86]. This pathway is crucial for embryonic development, but also remains active in regulation of adult stem cells responsible for maintaining and regenerating adult tissues, post-injury [87]. Abnormal overactivation of the Hedgehog pathway has been suggested to lead to tumorigenesis, through the transformation of adult stem cells into the cancer stem cells that give rise to tumors. When aberrantly reactivated, this developmental pathway has been implicated in multiple human cancers, including MPM [88,89,90].

In a study of MPM specifically, in which quantitative PCR and in situ hybridization were used on 45 clinical samples, *GLI1*, *SHH*, and human hedgehog interacting protein (*HHIP*) gene expression were significantly increased in MPM tumors, compared with healthy pleural tissue, although other Hedgehog pathway genes, such as *PTCH1*, *IHH*, *DHH*, *SMO*, and *GLI2* did not differ [91]. In that study, smoothened inhibitors suppressed hedgehog signaling in primary MPM cell culture systems and decreased tumor growth in MPM xenografts, indicating a potential therapeutic approach [91]. In contrast, we showed that elevated *SMO* expression levels strongly correlated with poorer overall MPM patient survival (*n* = 46), underscoring the heterogeneity of Hh pathway gene expression profiles in these tumor microenvironments [92]. Furthermore, whereas autocrine Hh pathway up-regulation was initially described in MPM pathobiology, Meerang et al. detected paracrine activation of Hh signaling in MPM patients and reported heterogeneous expression of *GLI1* in both tumor and stroma fractions [93]. Their study provides evidence for targeting Hh signaling, as well as a number of MPM-specific pathway perturbations in the stromal compartment, as a treatment strategy for MPM [94].

Additional heterogeneity in MPM tumors is introduced by Hh-independent Gli activation and we showed aberrant *GLI1* and *GLI2* activation in about 90% of MPM tissue samples (*n* = 46) [95]. We also demonstrated that targeting Gli using siRNA and small molecule inhibitors (alone and in combination with chemotherapy) suppressed cell growth dramatically, both in vitro and in vivo, providing strong support for Gli being a novel clinical target for MPM treatment [95]. Evidence suggests that crosstalk between Gli and other cell signaling pathways that are independent of Hh, such as Kirsten rat sarcoma (KRAS), EGFR and mTOR, are linked to worse clinical outcomes in MPM and crosstalk between Gli and mTOR actually causes increased Gli activation [96]. Finally, loss of the tumor suppressor Numb, known to regulate Notch, Hedgehog and TP53 pathways, was reported in ~50% of tissue specimens and has been associated with poor prognosis in epithelioid MPM [97]. Forced expression of Numb conferred sensitivity to cisplatin and activated apoptotic pathway proteins, suggesting potential therapeutic options for MPM [97].

Pharmacological inhibition of the Hh pathway could prove extremely useful as a therapeutic strategy. Most experimental models report Hh antagonists that target Smo or Gli, such as cyclopamine, vismodegib, GANT61, arsenic trioxide or GLI-I, strongly suppressed MPM cell viability by blocking Gli activation. Several clinical trials of Hh inhibitors are underway; thus far, three mesothelioma patients who participated in the phase I study for vismodegib have not responded to treatment [94], but their tumors were not assessed for Hh activity before study enrollment. Thus, reliable biomarkers and patient stratification would significantly ameliorate unfavorable clinical outcomes so that appropriate individuals who can benefit most are selected [95,98].

### 5.2. Wnt/β-Catenin Pathway

The Wnt signal transduction pathway is activated by a secreted Wnt ligand, from a 19-member family, binding to one of 10 cell-surface Frizzled (Fzd) receptors and to LRP5/6 [99,100]. Through a series of intracellular events in the canonical pathway, Fzd signals to the Dishevelled (Dvl) proteins, which causes an accumulation of β-catenin in the cytoplasm, followed by nuclear translocation and activation of the T-cell factor/lymphoid enhancer factor (TCF/LEF) family of transcription factors in a context-dependent manner [99,100]. In the absence of Wnt, β-catenin is degraded in the cytoplasm via a destruction complex (Axin, APC, GSK3, PP2A, CK1α) and the pathway becomes inactive [99,100]. This conserved pathway orchestrates cell fate decisions during embryonic development, maintenance of self-renewing stem cells in adults, and integration of signaling cues from other pathways, like bone morphogenetic protein (BMP), FGF, TGF-β and retinoic acid [99]. Mutations in the Wnt pathway are frequently observed in cancer, including germline mutations in *APC*, responsible for familiar adenomatous polyposis and colorectal cancers [101]. Loss-of-function mutations in Axin lead to hepatocellular carcinomas, and mutations in β-catenin lead to melanoma and several solid tumors; in addition, studies have reported the expression of β-catenin, a Wnt stem cell pathway activation indicator, in the progression of mesothelioma [102,103].

In a comparative Wnt-specific microarray study between MPM and normal lung tissue, our laboratory reported that the most common event in MPM was up-regulation of Wnt2 [104]. Subsequent targeted-Wnt2 inhibition via siRNA and a monoclonal Ab has an anti-proliferative effect and prompted apoptosis in MPM cells, thereby validating Wnt2 as a therapeutic target [104,105]. Other members of the Wnt signal transduction pathway, such as secreted frizzled-related proteins (sFRP), which are negative Wnt modulators, were shown to be transcriptionally down-regulated in MPM primary tissues and cell lines [106].

To identify meaningful biomarkers in MPM, our group used qRT-PCR to evaluate the expression of Wnt7A in 50 tumor specimens from patients who underwent surgical resection at the University of California, San Francisco (UCSF) [107]. We found that Wnt7A expression predicted sensitivity to chemotherapy; in particular, low Wnt7A expression was significantly correlated with negative overall survival in a univariate analysis, and patients with epithelioid tumors and low Wnt7A had significantly worse prognoses [107]. Furthermore, patients who had low Wnt7A-expressing tumors and received neoadjuvant chemotherapy had a better prognosis than those who did not [107]. Finally, a set of microRNAs was identified, which antagonize Wnt signaling, and were all down-regulated in MPM, compared to lung adenocarcinoma; these biomarkers could facilitate differential diagnoses between these different, but related, cancers [108].

About two dozen small-molecule inhibitors have been designed to target the Wnt pathway, but the most effort has been aimed at the TCF/β-catenin complex, to try to block signaling at the transcriptional level [101]. Of these inhibitors, LGK-974, a protein-serine *O*-palmitoleoyltransferase porcupine (PORCN)-specific inhibitor that is a key regulator of the Wnt signaling pathway, is currently being evaluated in phase I clinical studies for solid malignancies with documented genetic alterations upstream in the Wnt pathway, but no MPM patients were reported to have enrolled (NCT01351103). Additional small molecules have been developed for other Wnt pathway targets, such as β-catenin, GSK-3β, KCNQ1/KCNE1, Wnt3A, TNKS1/2, TNIK, and Axin/β-catenin interaction. However, it is not yet known whether any of these or other therapies that target the Wnt pathway will be effective in curbing the growth of MPM tumors.

### 5.3. Notch Pathway

Yet another stem-cell signaling and/or developmental pathway that exerts its effect on MPM cell lines, is the Notch pathway, which maintains tissue homeostasis and regulates neural stem cells in adults [109,110]. Four single-pass transmembrane receptors—Notch 1–4—consisting of large extracellular domains, get bound to a Notch ligand, which induces proteolytic cleavage and release of the intracellular domain, which translocates to the nucleus to influence gene transcription [111]. The Notch family plays an important role in normal development, and when it goes awry, promotes tumorigenesis [112]. As described in the Hh pathway, Numb acts as a tumor suppressor and in a Notch-specific context, promotes neural differentiation and maintains stem cell compartments; loss of Numb expression is linked to worse clinical prognoses in epitheliod MPM, and its upregulation could be associated with chemosensitivity, making it an informative biomarker [97].

Paralog-specific heterogeneity in the Notch pathway has been reported, since Notch1 and Notch2 exhibit opposing effects, depending upon the disease context [113]. For instance, Notch2 suppresses the effects of Notch1 in mesothelioma, but in medulloblastoma, Notch2 actually stimulates tumorigenesis, whereas Notch1 inhibits it [110]. Furthermore, several Notch ligands exist, and some trigger pathway activation, whereas others have inhibitory effects; thus, the complexities and biological consequences need to be carefully considered in therapeutic targeting [114,115]. Several small molecules have been developed to target Notch and γ secretase and a few have advanced to phase II/III clinical trials for indications like solid cancers and Alzheimer’s disease; testing these molecules in MPM might be worthwhile [116,117]. We have shown that CK2α inhibition down-regulates Notch1 signaling and reduces CSC-like populations in human lung cancer cells. Since there are no active trials of CK2 inhibitors in mesothelioma, small-molecules like CX-4945 or CIGB 300 anti-CK2 peptides may have therapeutic utility [118]. Interestingly, CK2 participates in the regulation of Hedgehog (Hh)/Gli1 signaling, underscoring the prevalence of cross-talk between crucial stem cell pathways implicated in cancer [119]. However, since much less investigation of MPM and Notch signaling has been reported, as compared to Hh and Wnt, further studies are warranted.

### 5.4. Hippo/YAP Pathway

Hippo signaling regulates organ size, cell contact inhibition, and stem cell self-renewal, but dysregulation can result in cancer development [120,121]. Central to Hippo signaling is a cascade of kinases, comprising Mst1/2, which complex with SAVI to phosphorylate and activate LATS1/2 [120]. These phosphorylation events inhibit two major downstream effectors of the Hippo pathway–YAP and TAZ—to stymie signaling [120,122]. However, in the dephosphorylated state, YAP/TAZ interact with TEAD 1–4 and other transcription factors in the nucleus to activate cell proliferation genes and inhibit apoptosis [120]. The Hippo pathway is regulated by several upstream factors, and two in particular, the *NF2*/*Merlin* and *YAP* oncogenes, are mutated or overactive in approximately 40% and 70% of MPM cases, respectively [96]. Furthermore, *NF2* regulates homeostasis in tissue repair and controls stem cell signaling, making it the most notorious perpetrator of MPM oncogenesis and an ideal therapeutic target for drug development [96]. In fact, Bueno et al. performed integrated analyses on 216 MPM samples and identified alterations in Hippo, among the most frequent signaling pathways in MPM [123].

In an effort to group MPM patients by molecular sub-type, to address intertumoral heterogeneity, Tranchant et al. reported that the tumor suppressor *LATS2* gene was altered in 11% of MPM [124]. They identified a new subgroup, C2LN, in which mutations in the *LATS2* and *NF2* genes co-occur within the same MPM and these patients showed enhanced sensitivity to PF-04691502, a PI3K-AKT-mTOR inhibitor [124]. Also, these investigators reported the *MOK* gene as a specific potential biomarker, noting that studies like these would be ideal for ameliorating clinical outcomes based on specialized gene signatures, but no clinical trial information is available [124]. To develop targeted treatment for *NF2*-mutant cancers, Cooper et al. demonstrated that MLN4924, an NEDD8-activating enzyme (NAE) inhibitor, suppresses CRL4DCAF1 (E3 ubiquitin ligase) and interferes with YAP activation in *NF2*-mutant cancers alone, and in combination with, the PI3K-mTOR inhibitor, GDC-0980 [125].

In order to investigate the association between *NF2*/*Merlin* and *YAP* gene alterations and clinical outcomes, Meerang et al. performed multivariate analyses in MPM patients [126]. They found that NF2/Merlin protein expression and the Survivin labeling index were prognosticators for poor clinical outcomes in two independent MPM cohorts, indicating that this data could guide treatment selection [126]. Interestingly, Felley-Bosco et al. reported an interaction between Hedgehog and YAP signaling, which makes Hh-specific modulating agents amenable to treating MPM [96]. Therapeutic strategies, aimed at disrupting YAP activity, include (1) targeting the Hh pathway as it down-regulates the YAP protein, (2) a small molecule called verteporfin, which inhibits YAP-TEAD transcription factor assembly, and (3) obstructions of lysophosphatidic acid (LPA) and thrombin receptor signaling, because upon agonist binding, they activate YAP [96].

We have outlined a few stem cell pathways which not only get deregulated in several types of cancer, but also in MPM. A number of therapeutics have been developed for components of these pathways, which are under clinical investigation and may be used for patient care in the near future.

## 6. Conclusions and Perspectives

Over the past 10 years our knowledge of MPM biology has advanced substantially, specifically regarding the molecular pathways involved, genetic and epigenetic associations, and the heterogeneous immune microenvironment of MPM tumors. This progress has led to the development of new and exciting therapeutic strategies and treatment options for patients diagnosed with this deadly malignancy. However, a “one-size fits all” approach will not suffice because heterogeneity in the tumor microenvironment exists from one malignancy to another, and one patient to another, and also evolves in response to therapies administered. In addition, stratification of patients based on genetic signature to guide targeted therapeutic interventions, such as the use of CDK and mTOR inhibitors, and epigenetic modulators, could be more effective for treating patients. Furthermore, several clinical investigations of therapies outlined in this review for MPM are underway.

## Figures and Tables

**Table 1:** Malignant Pleural Mesothelioma (MPM) Targets, Potential Therapeutics, and Clinical Status.

Pathway	Target(s)	Prevalence of Target in MPM (%)	Therapeutic	Clinical Trial Status
Angiogenesis	VEGF	30% expression	Bevacizumab	Approved for colon cancer
	VEGF	30% expression	Bevacizumab + pemetrexed and cisplatin	Clinical trial phase III completed
	VEGFR	20% expression	Dovitinib; nintedanib; cediranib	Under clinical investigation
	VEGFR	20% expression	Vatalanib	Under clinical investigation
	VEGF, FGF	30%, 50% overexpression	Lenvatinib	Approved for thyroid/kidney Cancers
	HGF	85% overexpression	Adenovirus containing an HGF variant, NK-4	-
	?	?	Thalidomide	Approved for multiple myeloma
	VEGFR, PDGFR	20%, 30% overexpression	Sorafenib	Approved for thyroid, kidney, and liver cancer
	VEGFR, PDGFR	20%, 30% overexpression	Sunitinib	Approved for kidney and GI cancer
Apoptosis	p53	20–25% mutated	-	-
	*Survivin*	-	Antisense oligonucleotides + chemotherapy	-
	Bcl-xL	-	Small molecule HDAC inhibitors + antisense oligonucleotides	-
	Bcl-xL/Bcl-2	-	2-Methoxy antimycin A3 + chemotherapy	-
	Src	~50% expression	Dasatinib	Approved for leukemia
	Fas Ligand	Selective FasL-positive cells	Fas ligand + cisplatin	-
	Calcium Channels	Primary samples showed reduced calcium ion uptake	Exgogenous calcium ions or mitochondrial calcium uniporter	-
	TRAIL	-	Administration of MSCs genetically engineered to express TRAIL	-
Cell Cycle	*CHEK1*	50% overexpression	*CHEK1* silencing + doxorubicin	-
	*YAP*	~70% expression	*YAP* silencing	-
Growth Factor	EGFR	~40% Expression	Gefitinib; Erlotinib	-
	*PDGFR*	20%, 30% overexpression	*PDGFR* silencing	
	FGFR1	50% overexpression	FGFR-1 inhibitor PD-166866	-
	FGFR1	50% overexpression	Sorafenib	Approved for thyroid, kidney, and liver cancer
	FGFR1	50% overexpression	Ponatinib	Approved for thyroid, kidney, and liver cancer
DNA Replication	TERT	90% overexpression	Anti-telomerase drugs + other targeted therapies	-
Tumor Suppressor-associated targets	CDK2	70% homozygous deletion of *CDKN2A* known to inhibit CDK2	Milciclib/PHA-848125AC	Phase II for hepatocellular carcinoma
	Snail-p53	30–45% *NF2* deletion known to prevent p53 inhibition	GN25	-
	EZH2	60% *BAP1* inactivations known to modulate EZH2	Pinometostat (EPZ5676) and other methyltransferase inhibitors	-
Stromal Compartment	PD1	(%?) tumor microenvironment is immunosuppressive	Nivolumab	Phase II for MPM
Hedgehog	Smoothened	Inhibits Hh signaling (%?)	Vismodegib	Approved for basal cell carcinoma
	Gli	90% Gli1/Gli2 active	GANT61 and GLI-I	-
Wnt/β-catenin	PORCN	Inhibits Wnt signaling (5%)	LGK-974	Phase I for solid tumors
Notch	γ secretase	Inhibits Notch signaling (%?)	Semagacestat (LY450139)	Phase III for Alzheimer’s disease
	CK2α	Down-regulates Notch1 signaling (%?)	Silmitasertib (CX-4945)	Phase I for solid tumors and multiple myeloma
Hippo/YAP	PI3K-AKT-mTOR	*LATS2* altered in 11%	PF-04691502	-
	Nedd8 activating enzyme (NAE)	Interferes with *YAP* (70%) activation	Pevonedistat (MLN4924)	Phase I for hematological malagnancies and melanoma
	YAP-TEAD	*NF2* (40%) and *YAP* (70%) overactive	Verteporfin	Phase I for prostate cancer

Note: - indicates no data and ? indicates unknown. The above-mentioned therapeutics have not yet been approved for MPM but some of them could be promising in treating it in the near future.
